# Supplementary material for: MicroRNA-210 Enhances Cell Survival and Paracrine Potential for Cardiac Cell Therapy While Targeting Mitophagy
Source: J Funct Biomater. 2025 Apr 21;16(4):147. doi: 10.3390/jfb16040147 (PMC12028018; doi:10.3390/jfb16040147)
Supplement: Supplementary file 1 [file jfb-16-00147-s001.zip › jfb-3494546-supplementary.pdf]

Journal of
Functional Biomaterials

MDPI

Article

MicroRNA-210 enhances cell survival and paracrine potential for cardiac cell therapy while targeting mitophagy

Rita Alonaizan [1,2,*], Ujang Purnama [1], Sophia Malandraki-Miller [1], Mala Gunadasa-Rohling [1], Andrew Lewis [2], Nicola Smart [1] and Carolyn Carr [1]

1 Department of Physiology, Anatomy & Genetics, University of Oxford, Oxford, UK
2 King Faisal Specialist Hospital & Research Centre, Riyadh, Saudi Arabia
* Correspondence: rita.al-onaizan@hotmail.com; rita.alonaizan@dpag.ox.ac.uk

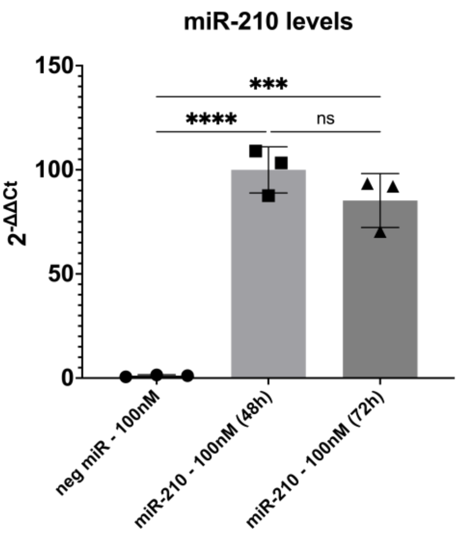

Figure S1. Confirmation of miR-210 overexpression after transfection in CPCs. miR-210 levels in CPCs measured by qRT-PCR at 48 and 72 hours following DharmaFECT transfection with miR-210, compared to CPCs transfected with the negative miRNA. Analysed using a one-way ANOVA with Tukey's multiple comparisons test (n=3).

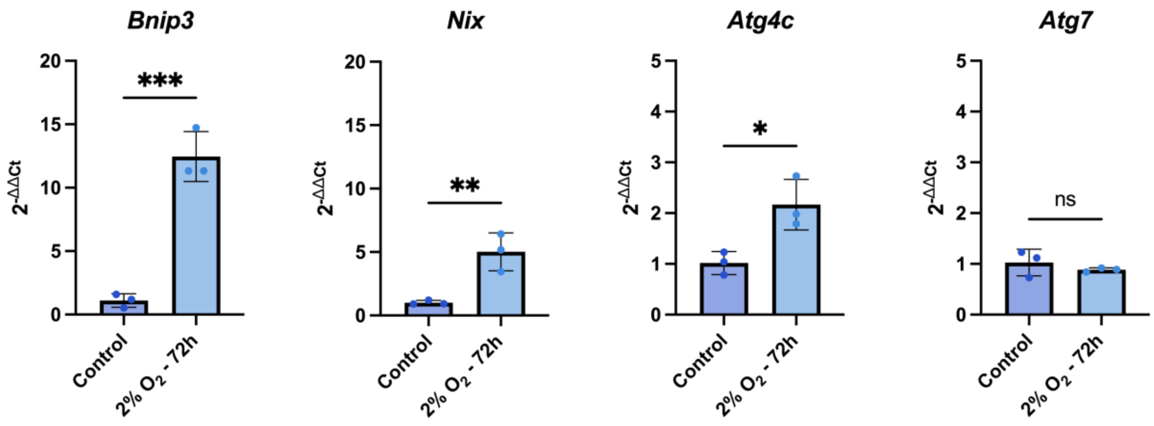

Figure S2. Hypoxic culture upregulates the mRNA levels of mitophagy-associated genes in CPCs. *Bnip3*, *Nix*, *Atg4c* and *Atg7* mRNA levels in CPCs cultured in hypoxia, in comparison to CPCs cultured in normoxia. Analysed using a t-test (n=3).

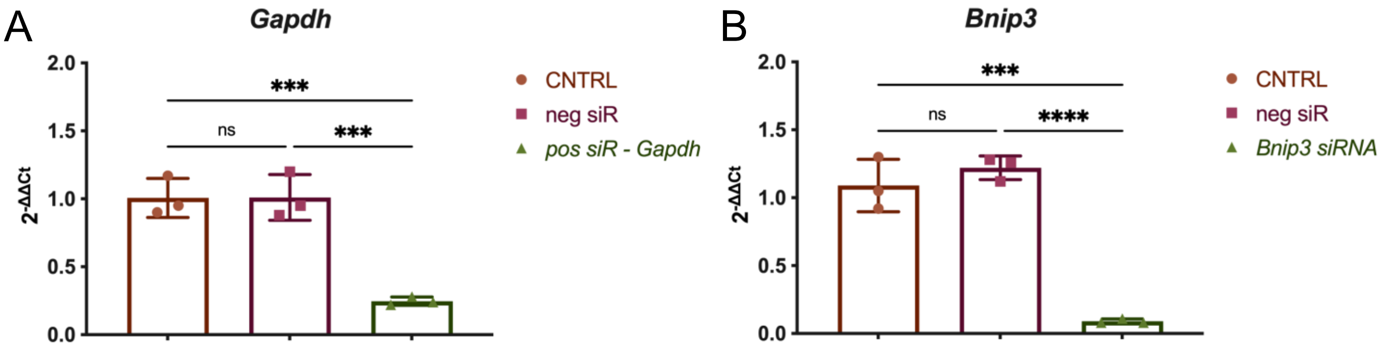

Figure S3. Validation of *Bnip3* siRNA. (A) *Gapdh* mRNA levels following transfection of CPCs with a positive control *Gapdh* siRNA. (B) *Bnip3* mRNA levels following transfection of CPCs with *Bnip3* siRNA at 100nM in comparison to a negative siRNA and an untransfected control. Analysed using a one-way ANOVA with Tukey's multiple comparisons test (n=3).

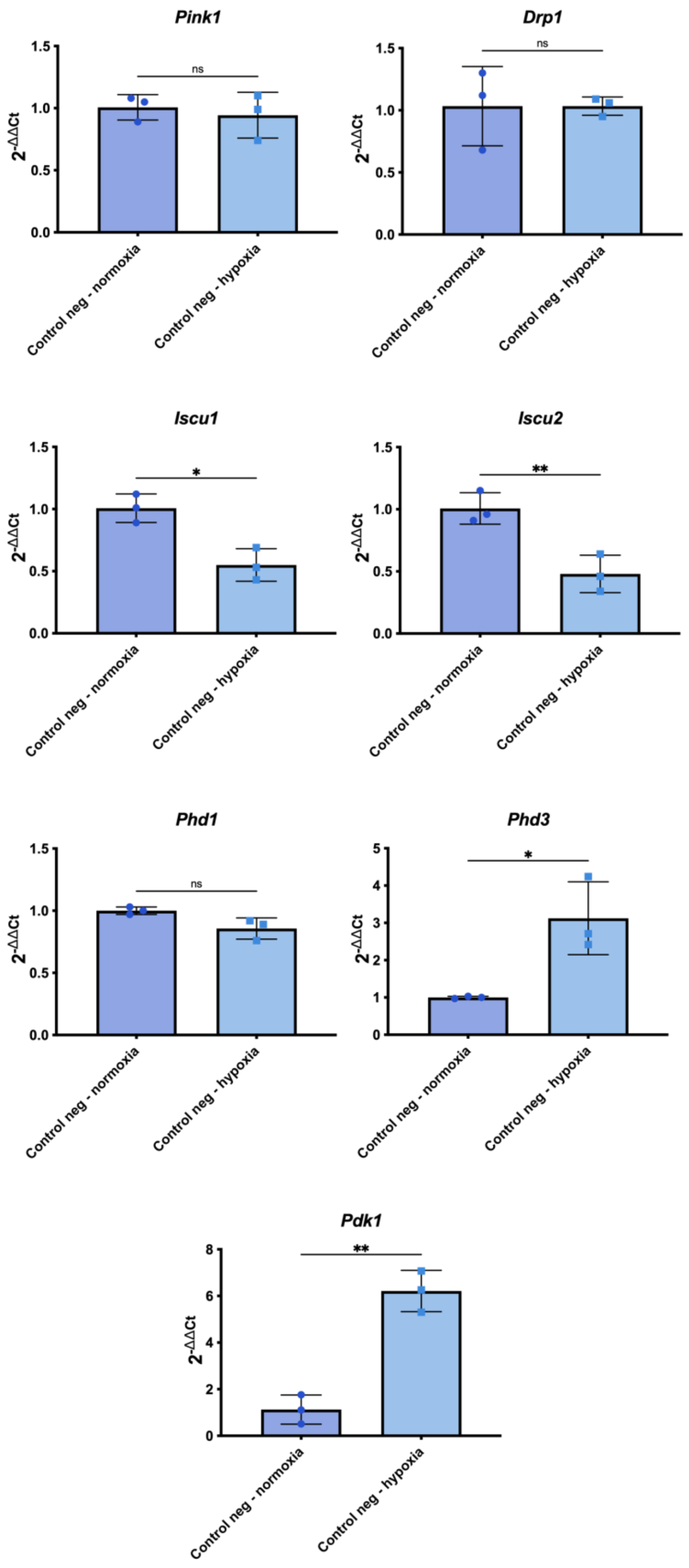

Figure S4. Expression of mitophagy- and hypoxia-associated genes in CPCs cultured in hypoxia. mRNA levels of mitophagy- and hypoxia-associated genes in CPCs transfected with a negative miRNA and cultured in hypoxia, in comparison to CPCs transfected with a negative miRNA and cultured in normoxia. The data are recycled from previous figures and were therefore added to the supplementary material. Analysed using a t-test (n=3).

Table S1. qRT-PCR primers.

Target	Forward sequence	Reverse sequence	Reference
Hprt	TCAGTCAACGGGGGACATAA	GGGGCTGTACTGCTTAACCAG	[81]
Atg4c	GGAGCCTCTCTCGATTCTGC	CCAATATAAGTTCAAAGGGCGAGG	NCBI Ref Seq: NM_001145967.1
Atg7	TGGAGTTCAGTGCTTTTGAC	GGTGTTGTGCAGGGTTCC	[82]
Bnip3	CAGCATGAATCTGGACGAAG	ATCTTCCTCAGACAGAGTGC	[83]
Casp8ap2	GATGTCTGTCCTGCCTCTCC	AACAGCACTGTCCAACCCAG	NCBI Ref Seq: NM_001122978.2
Drp1	ATGCCAGCAAGTCCACAGAA	TGTTCTCGGGCAGACAGTTT	[84]
Iscu1	ACCACAAGAAGGTTGTGGATCA	CACCAATCCGGTTCCAACAT	NCBI Ref Seq: NM_025526.5
Iscu2	CGAACCATCGAGGGGACC	CATAATGATCCACAACCCGGAC	NCBI Ref Seq: NM_001363317.1
Nix	CCTCGTCTTCCATCCACAAT	GTCCCTGCTGGTATGCATCT	[85]
Pdk1	GGCGGCTTTGTGATTTGTAT	ACCTGAATCGGGGGATAAAC	[86]
Phd1	TTGCCTGGGTAGAAGGTCAC	GCTCGATGTTGGCTACCACT	[86]
Phd3	CAACTTCCTCCTGTCCCTCA	GGCTGGACTTCATGTGGATT	[86]
Pink1	GCTGGTGAGGTCACTGCTC	TCTTCAGGGCTAGAAGATGCTC	[87]
mt-Nd1	GAGCATCTTATCCACGCTTCC	GGTGGTACTCCCGCTGTAAA	[88]
B2m	CACAGTTCCACCCGCCTCACA	TGGGCTCGGCCATACTGGCA	[88]
miR-210	Mm_miR-210_2 miScript Primer Assay ThermoFisher product no. 218300		MS00032564
Snord68	Hs_SNORD68_11 miScript Primer Assay ThermoFisher product no. 218300		MS00033712
